# Comparison of Long-Term Atmospheric Aging between *Fe*_3_ *O*_4_ and PEG-Protected *Fe*_3_ *O*_4_ Nanoparticles

**DOI:** 10.1021/acsomega.5c08246

**Published:** 2025-11-05

**Authors:** Ali Erdoğan, Olcay Kizilaslan

**Affiliations:** Department of Biomedical Engineering, Faculty of Engineering, 37520 Inonu University, Malatya 44280, Turkey

## Abstract

Magnetic nanoparticles have attracted significant attention due to their broad range of applications. A considerable amount of research has been conducted on the oxidation of Fe_3_O_4_ nanoparticles; however, there remains a significant lack of systematic data on the long-term oxidation mechanisms of these nanoparticles under ambient atmospheric conditions. A comprehensive one-year study was conducted to investigate the magnetic properties of uncoated and PEG-coated Fe_3_O_4_ nanoparticles. The systematic magnetization versus magnetic field measurements were performed over one year, with the samples stored under ambient atmospheric conditions and measured at regular intervals of a few weeks. To further characterize the synthesized nanoparticles, XRD, FTIR, and DLS analyses were conducted. The time-dependent oxidation process was modeled using an exponential function, and the fitting parameters were analyzed to provide physical insight into the oxidation behavior. The findings offer valuable perspectives on the potential applications of Fe_3_O_4_ nanoparticles, particularly in magnetically driven technologies across various fields, while also providing a deeper understanding of the slow oxidation mechanisms of uncoated and PEG-coated Fe_3_O_4_ nanoparticles.

## Graphical abstract

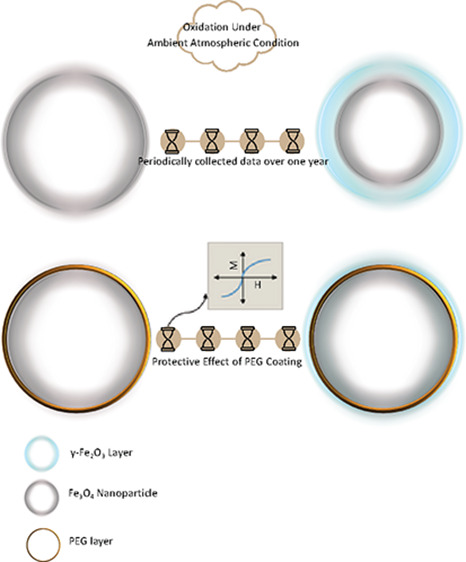

## Introduction

There is a growing demand for magnetic nanoparticles (MNPs) across various fields of research and technology.^1–6^ MNPs have attracted significant attention as high-performance anode materials for supercapacitors in energy storage applications.^7–10^ They have been employed in mineral processing and wastewater treatment,^11,12^ as well as in various biomedical in vivo applications, including hyperthermia treatment,^13^ drug delivery,^14–17^ and as contrast agents for magnetic resonance imaging (MRI).^18–22^ Factors such as temperature, pH, and iron ion concentration significantly influence the size, morphology, and composition of MNPs.^21,23–27^ To date, numerous methods for synthesizing MNPs have been developed and continuously refined.^24,28,29^

Iron ions (Fe^2+^ and Fe^3+^) are highly versatile in forming various oxide and (oxy)­hydroxide structures due to their ability to adopt different oxidation states. This versatility, combined with environmental factors such as pH, temperature, ionic strength, and oxygen availability, results in the crystallization of more than 20 distinct iron oxide and (oxy)­hydroxide forms, including well-known compounds like magnetite (Fe_3_O_4_), hematite (Fe_2_O_3_), goethite (α-FeOOH), and lepidocrocite (γ-FeOOH).^30^ Different iron oxide and (oxy)­hydroxide materials are also known to transform into one another, a process that occurs more rapidly in nanoparticles (NPs) compared to bulk materials.^31^ NPs have a much higher surface area-to-volume ratio compared to bulk materials. This increases the surface energy, making the NPs thermodynamically unstable and more reactive, thus accelerating phase transformations. A considerable amount of research has been conducted to explore and understand the oxidation mechanisms of Fe_3_O_4_ NPs;^30,32–34^ however, information on the long-term stability of their magnetic properties remains limited.

A decrease in the magnetization of MNPs over time can lead to various issues, particularly in magnetically driven applications across different fields. In drug delivery systems, it could result in lower targeting precision, as diminished magnetization might reduce the ability to guide NPs using an external magnetic field.^35^ Similarly, in hyperthermia treatment, decreased magnetization can lower heat generation efficiency, thereby diminishing therapeutic effectiveness.^36^ In MRI, reduced magnetization could decrease contrast, possibly leading to lower image clarity.^37^ In water treatment, it might make the separation processes less efficient.^38^ In data storage, it could increase the likelihood of data loss or reduced storage density.^39,40^ Furthermore, it may reduce the efficiency of recovering catalysts using magnetic separation, thereby affecting their reusability.^41^ In magnetic actuators or sensors, decreased magnetization could impair sensitivity and responsiveness, potentially diminishing performance.^42^

The aim of this paper is to investigate the magnetization properties of Fe_3_O_4_ NPs as a function of oxidation over a one-year period under ambient atmospheric conditions. The experiments primarily involved magnetic measurements. The oxidation of stoichiometric Fe_3_O_4_ NPs over time resulted in an exponential decrease in magnetization. The decrease was observed as 33.05% for PEG-coated NPs and 11.05% for uncoated NPs after approximately a year, indicating reductions that could substantially impact device performance, especially in magnetically driven applications across diverse fields. This work is expected to provide valuable insights into the long-term stability of Fe_3_O_4_ NPs and offer guidance on their use in practical applications.

## Synthesis of NP

Fe_3_O_4_ NPs were synthesized using the coprecipitation method,^43,44^ where a solution containing a mixture of FeCl_2_ and FeCl_3_ was reacted with NaOH, leading to the formation and precipitation of Fe_3_O_4_. In a standard procedure, 10 mL of 1.5 M HCl was mixed with 3.98 g of FeCl_2_·4H_2_O and 10.81 g of FeCl_3_·6H_2_O. The mixture was dissolved in 400 mL of deionized water and heated to 50 °C in a beaker. During the synthesis process, nitrogen gas was vented into the environment. When the water temperature reached 50 °C, a 1.5 M NaOH solution was introduced into the synthesis environment by using a syringe pump at a rate of 60 mL/h. By controlling and maintaining the synthesis temperature, the pH was adjusted to 11. When the pH reached 11, the addition of NaOH was stopped, and the mixture was stirred for an additional 30 min under N_2_ gas while maintaining the synthesis temperature. Subsequently, the synthesized material was allowed to cool naturally over a period of 45 min. Then, the material was washed three times with deionized water. The washed material was diluted to 100 mL by adding deionized water and then dried at 65 °C for the characterization measurements.

The surfaces of the synthesized NPs were functionalized with silane compounds.^43^ A solution of 2.20 mL of 3-aminopropyltrimethoxysilane, APS (12.4 mmol), dissolved in 10 mL of methanol was added to a mixture consisting of 20 mL of ferrofluid, equivalent to 8.8 mmol of iron, and an additional 10 mL of methanol. The mixture was stirred at room temperature for 12 h. 20 mL of glycerol was added to the resulting solution, and the methanol was removed first, followed by water, using a rotary evaporator. Following the evaporation process, the solution was dried under vacuum conditions at 100 °C for a duration of 2 h. The treated NPs were washed three times by using 40 mL of a water/acetone mixture with a ratio of 30:70. Peptization was performed by adding 40 mL of water to the mixture and slowly reducing the pH to 3 with nitric acid with vigorous stirring. After silanization, the NPs were modified with polyethylene glycol (PEG). A mixture of methoxypoly­(ethylene glycol) 5000 propionic acid N-succinimidyl ester (activated PEG, 600 mg, 0.12 mmol) and 3.0 mL of silanized ferrofluids (containing 0.41 mmol of iron) was stirred in 15 mL of deionized water at room temperature for 24 h. The solution was purified through dialysis against water, with the process repeated every 4 h for a total of 5 cycles.

The experiments were conducted on PEG-coated and uncoated Fe_3_O_4_ NPs. Each group of the NPs was dried and subsequently placed on separate sample holders. Magnetic measurements were then performed periodically on both samples. The same samples were used for all magnetic measurements without being unmounted from the sample holders. The samples were stored under ambient atmospheric conditions. Magnetization vs magnetic field (M-H) measurements were conducted using a vibrating sample magnetometer (VSM) integrated with the Quantum Design PPMS-9T system. The crystal structure was analyzed through X-ray diffraction (XRD) using a Rigaku Miniflex 600 diffractometer with Cu–Kα radiation (λ = 1.5405 Å) over the angular range of 30° ≤ 2θ ≤ 80°. FTIR spectroscopy measurements were carried out using a PerkinElmer Spectrum One to examine the modification of the NPs by PEG molecules. The dynamic light scattering (DLS) measurements were conducted with a Malvern Zetasizer Nano ZS.

## Results and Discussion

Figure 1 presents the FTIR spectra of the PEG-coated Fe_3_O_4_ NPs, the uncoated Fe_3_O_4_ NPs, and the pure PEG. The peaks around 619 and 588 cm^–1^ are indicative of the Fe–O vibrations observed in both the PEG-coated and the uncoated Fe_3_O_4_ NPs, but not in the pure PEG, confirming the presence of the Fe_3_O_4_ NPs. A broad O–H stretching vibration around 3436 cm^–1^, a relatively sharp C–H stretching vibration around 2884 cm^–1^, and a sharp C–O stretching vibration around 1108 cm^–1^ are observed in both the PEG and the PEG-coated NPs. The peaks confirm the presence of the PEG residue in the final product; however, they are absent in the spectrum of the uncoated Fe_3_O_4_ NPs. This observation indicates that the presence of specific peaks in the FTIR spectrum, corresponding to the O–H, C–H, and C–O stretching vibrations, confirms that the PEG molecules have successfully coated the surface of the Fe_3_O_4_ NPs. Figure 2 depicts the hysteresis curve of the PEG-coated Fe_3_O_4_ NPs measured at 300 K during the first week. The particles exhibit a nearly zero coercivity, whereas the bulk material displays a coercivity value in the range of 115–150 Oe.^1^ This phenomenon is attributed to the superparamagnetic behavior of nanoscale magnetite particles. The saturation magnetization (*M*_S_) was determined from the fitting formula (eq 1) and found to be 62.24 emu/g, which is considerably lower than the bulk magnetization value of *M*_S_ (bulk) = 92 emu/g.^45^ All *M*_S_ values in this article were determined using the same method. In Figure 2, the magnetization curve was fitted to the Langevin function (eq 1) to validate the superparamagnetic behavior of the NPs at room temperature:

$$M=M_{S}\left(\coth\left(\frac{\mu H}{k_{B}T}\right)-\frac{k_{B}T}{\mu H}\right)$$ 1

where *k*_B_, μ, and *M*_S_ represent the Boltzmann constant, the intrinsic magnetic moment of each particle, and the saturation magnetization, respectively. From the fitting, the superparamagnetic behavior of the NPs at room temperature is clearly observed.

**Figure 1:**
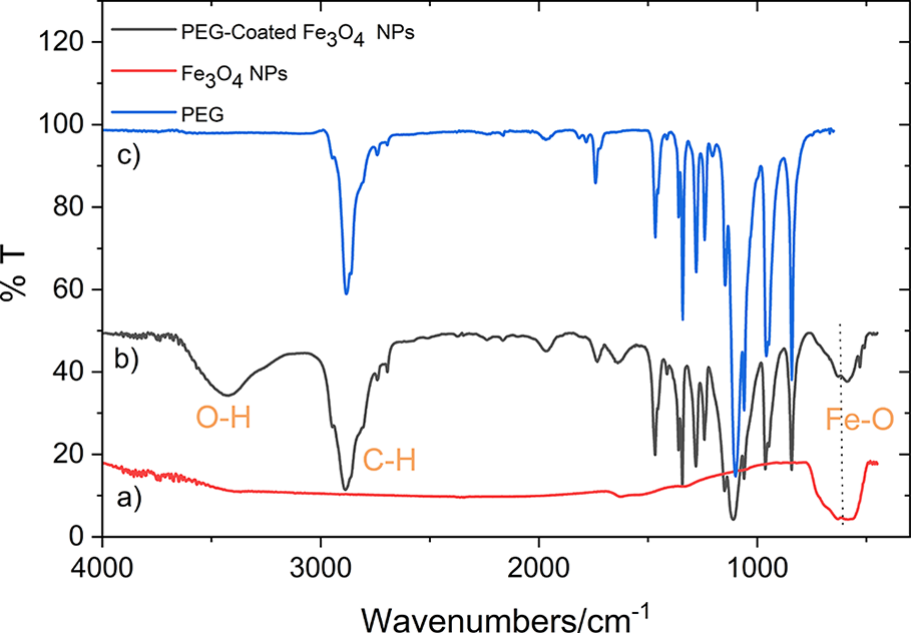
FTIR spectra of (a) the uncoated Fe_3_O_4_ NPs, (b) the PEG-coated Fe_3_O_4_ NPs, and (c) pure PEG.

**Figure 2:**
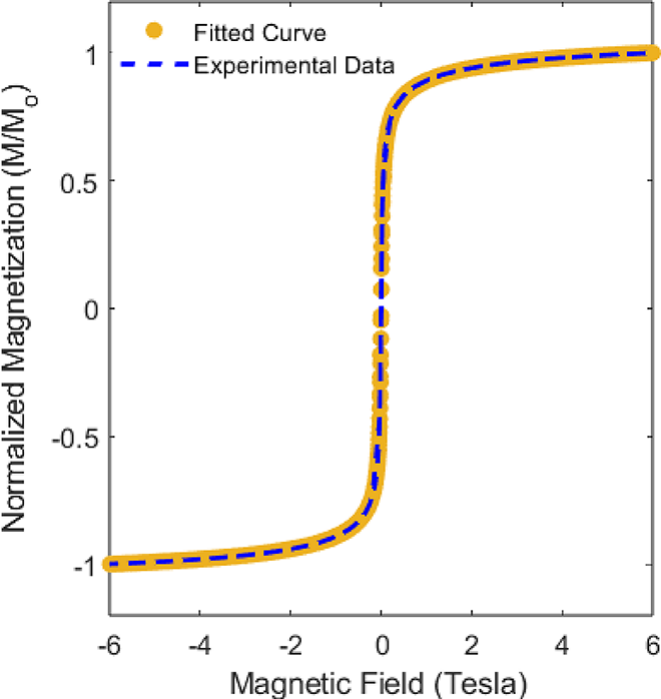
Magnetization vs magnetic field (M-H) curve of the PEG-coated NPs at 300 K, where Mo represents the magnetization value at 6 T.

Figure 3a illustrates the DLS measurements of the uncoated and PEG-coated Fe_3_O_4_ NPs measured during the first week. The peaks indicate that the NPs exhibit a high degree of monodispersity in size. The sharpness of the peaks emphasizes a highly uniform hydrodynamic diameter of approximately 8 nm for the uncoated NPs, suggesting a well-controlled particle size distribution. In contrast, the PEG-coated NPs show a larger hydrodynamic diameter of about 14.6 nm, which is consistent with the presence of the polymer shell. Additionally, the particle diameter, calculated from the M–H curve using the method described in ref 33, was found to be 7.72 nm. Figure 3b presents the FSEM image of the synthesized uncoated NPs, revealing that the particles are predominantly spherical in shape.

**Figure 3:**
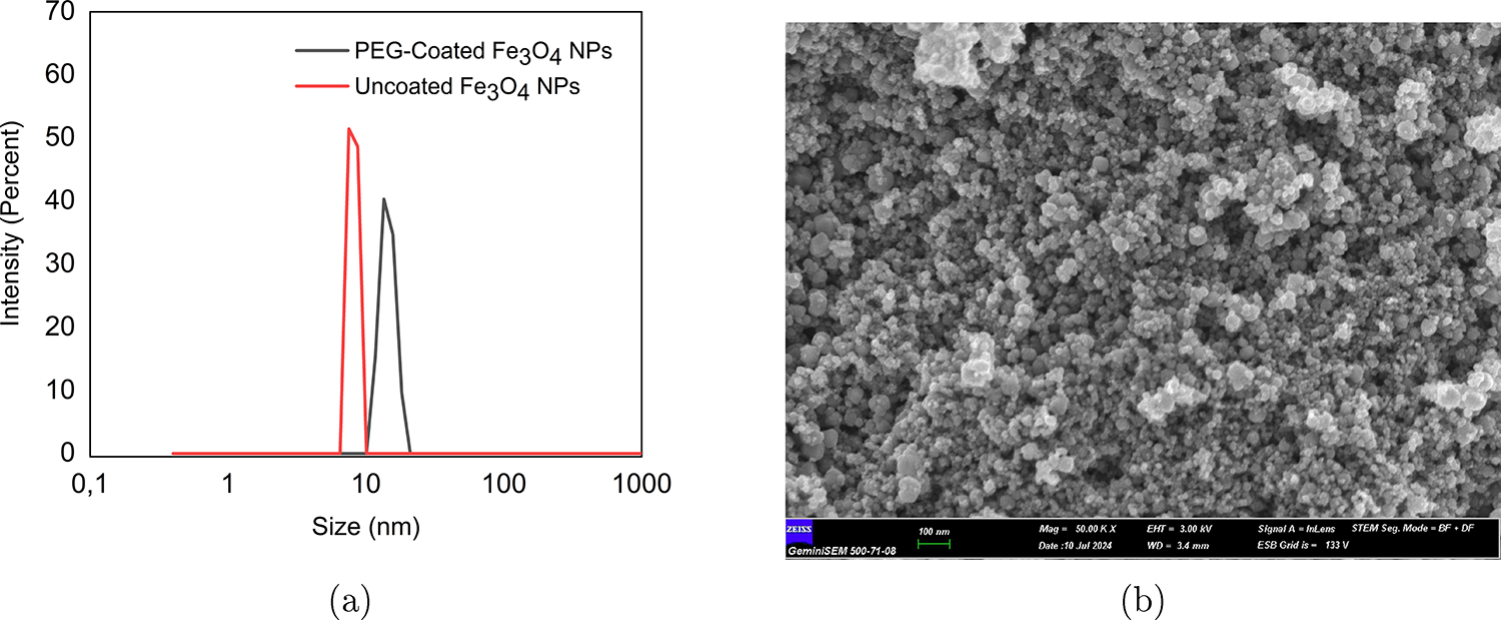
(a) Hydrodynamic size distribution of uncoated Fe_3_O_4_ nanoparticles measured by intensity. (b) FSEM image of the synthesized uncoated nanoparticles.

Figure 4 illustrates the decay of the saturation magnetization, *M*_S_, over a period exceeding one year for both the uncoated Fe_3_O_4_ NPs and the PEG-coated Fe_3_O_4_ NPs. The magnetic measurements were initiated 1 week after the synthesis; thus, the first data point represents the measurement taken during the first week. The PEG-coated Fe_3_O_4_ NPs exhibited higher saturation magnetization values compared to the uncoated Fe_3_O_4_ NPs, consistent with the findings reported in ref 46. The observed decay in magnetization is attributed to oxidation, likely resulting from storage under ambient atmospheric conditions following the drying process of the NPs. It is noted that in the dry state, the Fe_3_O_4_ NPs are readily oxidized to maghemite (γ-Fe_2_O_3_) by air even at room temperature, causing a topotactic reaction in which the original crystal morphology is preserved throughout the process.^31^ During the oxidation, it is known that a phase transition to a mixed phase (from magnetite to maghemite) takes place gradually, following the chemical formula Fe_1–x_^II^Fe_2+*x*_^III^O_4+0.5*x*_.^31^ The proportion of the magnetite phase decreases in relation to the extent of the oxidation. The divalent iron ions (Fe^2+^) at the surface of the magnetite, which are more prone to oxidize under ambient atmospheric conditions, are oxidized to Fe^3+^ ions.^30^ This process significantly impacts the chemical composition, magnetic properties, and overall stability of the Fe_3_O_4_ NPs, leading to a pronounced decay in magnetization, as observed in our study. Although not observed within the one-year time frame of this study, further oxidation is known to result in the transformation of the magnetite into hematite as represented by the following chemical reaction: 4Fe_3_O_4_ + O_2_ → 6Fe_2_O_3_. Such a complete phase transition from the Fe_3_O_4_ to γ-Fe_2_O_3_ may require higher temperatures to accelerate the oxidation process.^47^

**Figure 4:**
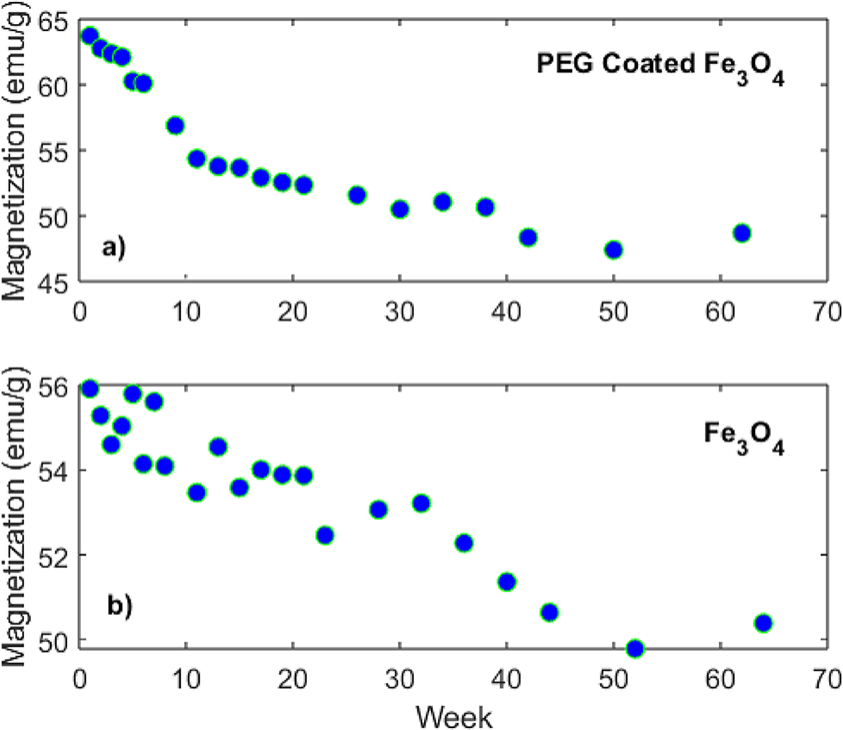
Saturation magnetization as a function of time (in weeks): (a) PEG-coated NPs and (b) uncoated NPs.

The data presented in Figure 4 were smoothed by using the moving average method to minimize noise and enhance the clarity of the underlying trends. This approach facilitated accurate interpretation while preserving the fundamental characteristics of the data set. The smoothed data are shown in Figure 5. The saturation magnetization as a function of time *t* was modeled using the equation *M*(*t*) = *a* · exp­(*t*/*b*) + *c*, where *a* represents the magnitude of the decaying magnetization, *b* denotes the time constant governing the decay rate, and *c* corresponds to the residual magnetization, reflecting the stable magnetization of the particle cores that remain unaffected by oxidation. The parameter *b* determines the rate at which the magnetization decays over time. A smaller absolute value of *b* corresponds to a faster decay/oxidation, while a larger absolute value of *b* indicates a slower decay/oxidation. The fitting parameters were determined to be *a* = 13.8, *b* = −125.1, and *c* = 41.67 for the uncoated NPs and *a* = 14.83, *b* = −14.74, and *c* = 48.88 for the PEG-coated NPs. Considering the *b*-value, the PEG-coated NPs exhibit faster oxidation compared to that of the uncoated NPs. In contrast, the PEG coating is generally known to protect the magnetic core from oxidation and agglomeration, suggesting a slower oxidation process. We believe that the primary reason for this discrepancy is that most of the oxidation occurred during the initial stages following the synthesis process. This observation is supported by the saturation magnetization values measured during the first week, which were lower for the uncoated NPs, indicating significant oxidation during the initial stages. The rapid oxidation of the uncoated NPs during this period likely resulted in the formation of oxidized surface layers, creating a protective barrier that subsequently hindered further oxidation by limiting oxygen diffusion into the core. Thus, we attribute the relatively slower long-term oxidation of the uncoated Fe_3_O_4_ NPs to the restricted diffusion of oxygen into the core. Based on the fit parameter *a*, the initial saturation magnetization of the PEG-coated NPs is greater than that of the uncoated ones. Similarly, the fit parameter *c* reveals that the residual magnetization of the PEG-coated NPs remains higher after one year.

**Figure 5:**
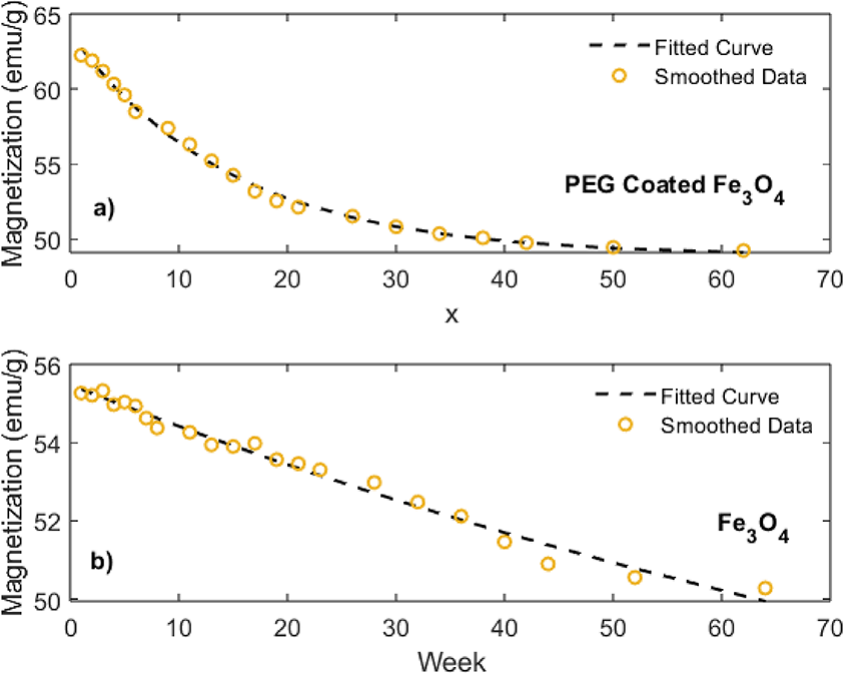
Smoothed saturation magnetization as a function of time (in weeks): (a) PEG-coated NPs and (b) uncoated NPs.

The structure of the iron oxide is analyzed using Mössbauer spectroscopy,^48–50^ Raman spectroscopy,^51^ and XRD.^52–56^ Although distinguishing between magnetite and maghemite using XRD is challenging^30,57^ due to their similar crystal structures and diffraction patterns, it can still provide valuable insights. To observe the structural changes in the Fe_3_O_4_ NPs over time, XRD measurements were conducted. However, unlike the magnetic measurements, these were not performed periodically. Figure 6a shows the XRD patterns of the uncoated Fe_3_O_4_ NPs measured during the first week and three years later, representing a longer time frame compared to our magnetic measurements. The XRD pattern of the PEG-coated NPs exhibited a weak signal due to the PEG layer, leading to a high error rate; therefore, it was excluded from further analysis. The NPs (after three years) show a noticeable decrease in the intensity of the diffraction peaks compared with the NPs measured during the first week. This suggests a reduction in the crystallinity of the Fe_3_O_4_, likely due to the oxidation and the surface structural modifications over time. The peaks labeled (220), (311), (400), (511), and (440) are consistent with the spinel structure of Fe_3_O_4_.^58^ Even after three years, these peaks are still present, suggesting that Fe_3_O_4_ remains the dominant phase, although its crystallinity has diminished. There are no additional peaks corresponding to γ-Fe_2_O_3_ (hematite), suggesting that the oxidation did not progress to the formation of a fully new crystalline phase. For further analysis, powder XRD peaks of the NPs, measured both in the first week and after three years of storage (220), (311), (400), (511), and (440), were fitted with pseudo-Voigt functions (Figure 6b). Using the Williamson–Hall (W–H) relation, $\beta \cos\;\theta =\frac{K\lambda }{D}+4\varepsilon \sin\;\theta$ , with Cu Kα radiation (λ = 0.15406 nm) and *K* = 0.89, we constructed plots of *y* = βcosθ versus *x* = 4sinθ, from which the intercept *a* = *K*λ/*D* and the slope *b* = ε were obtained.

**Figure 6:**
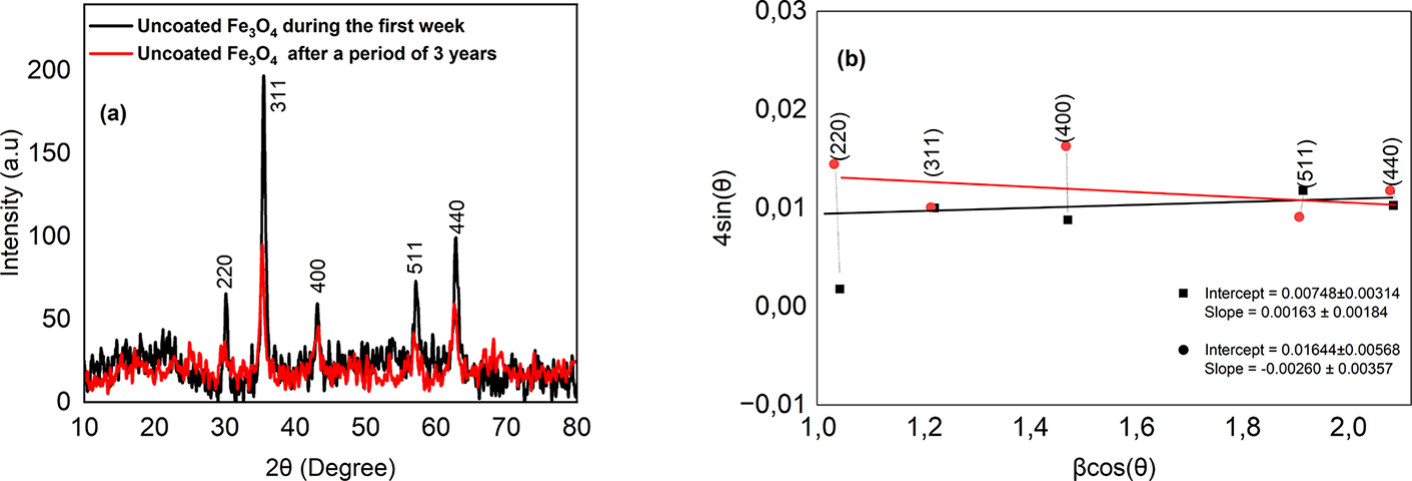
(a) XRD patterns of the uncoated Fe_3_O_4_ NPs measured in the first week and three years later. (b) Williamson–Hall plots for the uncoated Fe_3_O_4_ nanoparticles, recorded during the first week (square markers) and after three years of storage (circle markers), illustrating the evolution of crystallite size and microstrain.

For XRD peaks of the NPs measured in the first week, the linear fit yields a small, near-zero positive slope, indicating negligible microstrain within uncertainty; the intercept corresponds to a coherent crystallite size of *D*_0_ = 18.54 ± 7.78 nm. After three years of storage, the W–H slope becomes slightly negative and remains close to zero, again consistent with relaxed (low-strain) crystallites. The intercept increased, giving a reduced crystallite size of *D*_3*y*_ = 8.43 ± 2.91 nm. Thus, the coherent domain size decreases, while the strain term stays negligible. The peak-broadening behavior is therefore size-dominated at both times. We emphasize that this *D* is the XRD crystallite size (coherent diffracting domain) and is not expected to match hydrodynamic or TEM particle diameters; the observed reduction reflects internal fragmentation or disordering of the crystalline domains rather than a change in overall particle diameter. Considering the calculated measurement uncertainty, the XRD- and DLS-derived sizes are still consistent within error. The reduction in coherent crystallite size observed after three years of storage supports the interpretation that long-term oxidation induces structural disorder, fragmenting initially larger crystallites into smaller coherent domains without significantly altering the overall particle dimensions.

## Conclusions

The XRD and FTIR measurements confirmed the successful synthesis of both uncoated and PEG-coated Fe_3_O_4_ NPs, while DLS analysis demonstrated the monodispersity of the synthesized NPs. The comprehensive year-long study of the magnetic properties of Fe_3_O_4_ NPs under ambient atmospheric conditions revealed an exponential decay in saturation magnetization. The time-dependent oxidation process was modeled using an exponential function, with the fitting parameters analyzed to provide physical insight into the oxidation process. In particular, the fitting parameter *b* offered valuable information about the oxidation rate. The analysis of the fitting parameter *b* showed that the oxidation rate of the PEG-coated sample was relatively higher than that of the uncoated sample, resulting in a magnetization decay of 33.05% for the PEG-coated NPs compared to 11.05% for the uncoated NPs. The slower oxidation rate of the uncoated Fe_3_O_4_ NPs was attributed to their rapid oxidation in the initial stage immediately following the synthesis. Nevertheless, the residual magnetization remained higher for the PEG-coated NPs. Furthermore, the XRD measurements conducted to monitor structural changes in the uncoated Fe_3_O_4_ NPs after three years revealed a reduction in the crystallinity, likely due to oxidation and surface structural modifications over time.

## Abbreviations

NPs: MNPs, APS, PEG, DLS, XRD, FTIR, MRI
